# Bruceine a exerts antitumor effect against colon cancer by accumulating ROS and suppressing PI3K/Akt pathway

**DOI:** 10.3389/fphar.2023.1149478

**Published:** 2023-03-28

**Authors:** Chaozheng Zhang, Yuening Cao, Yi Zuo, Hongbin Cheng, Changqun Liu, Xila Xia, Bo Ren, Yun Deng, Maolin Wang, Jun Lu

**Affiliations:** ^1^ State Key Laboratory of Southwestern Chinese Medicine Resources, School of Pharmacy, Chengdu University of Traditional Chinese Medicine, Chengdu, China; ^2^ Department of Dermatology, Hospital of Chengdu University of Traditional Chinese Medicine, Chengdu, China; ^3^ Clinical Research Center, The First Affiliated Hospital of Shantou University Medical College, Shantou, Guangdong, China; ^4^ Department of Physiology, School of Basic Medical Sciences, Shenzhen University, Shenzhen, China

**Keywords:** Bruceine A (BA), colon cancer, network pharmacology, PI3K/AKT, molecular dock, apoptosis

## Abstract

Bruceine A (BA), a quassic ester from *bruceine javanica*, regulates diverse intracellular signal transduction pathways and manifests a variety of biological activities, however, its pharmacological mechanism in treating colon cancer (CC) is unclear. In this study, we investigated the anticancer effects of BA on CC cells and the underlying mechanisms. The network pharmacology research indicated that Akt1 and Jun and PI3K/Akt pathways are the predominant targets and critical signaling pathways, respectively, for BA treatment of CC. Meanwhile, molecular docking results implied that BA could conjugate to pivotal proteins in the PI3K/Akt pathway. BA remarkably suppressed the proliferation of CC cells HCT116 and CT26 with 48-h IC50 of 26.12 and 229.26 nM, respectively, and the expression of p-PI3K/p-Akt was restrained by BA at the molecular level as verified by Western blot assay. Further mechanistic studies revealed BA impacted cell cycle-related proteins by regulating the expression of P27 (a protein bridging the PI3K/Akt signaling pathway with cycle-related proteins), arresting the cell cycle in the G2 phase, inhibiting the proliferation of HCT116 and CT26, and facilitated the apoptosis in CC cells by activating the mitochondria-associated apoptosis protein Bax and accumulating reactive oxygen species, in addition to BA apparently inhibited the migration of CC cells. Taken together, our results demonstrated that BA might be a promising chemotherapy drug in the treatment of CC.

## Graphical Abstract

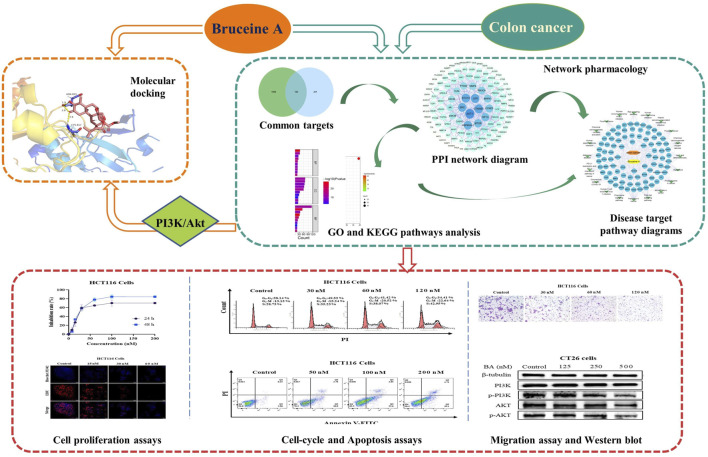

## Introduction

Colon cancer (CC) is one of the most prevalent gastrointestinal malignancies, with the third highest incidence and the fourth highest mortality rate in the world, as well as increasing morbidity in the last decade (Siegel et al., 2020). Currently, the conventional surgery is the primary treatment for CC, whereas radiotherapy, chemotherapy, and drug combination therapy are frequently adopted main treatments for advanced CC, yet recurrence, toxin accumulation, and drug resistance are the principal disadvantages of these treatments (Bagheri et al., 2018; Siddiqui et al., 2018; Van der Jeught et al., 2018; Wegner et al., 2020; Wang et al., 2021a; Wang et al., 2021b). An increasing amount of effective ingredients in traditional Chinese medicine (TCM) have been discovered and applied into various diseases (Amin et al., 2011; Li et al., 2017; Al-Dabbagh et al., 2018; Hamza et al., 2020; Pan et al., 2021; Ren et al., 2021; Chen et al., 2022; Sun et al., 2022; Zuo et al., 2022). Therefore, it is of significantly scientific and clinical implications to investigate the efficacy and mechanism of action of highly potent and low-toxic antitumor substances from TCM components on CC.

Bruceine A (BA) is a kind of active nigakilactone ingredient isolated from TMC Brucea *javanica* (Nakao et al., 2009). Studies have demonstrated that BA concurrently exerts a multitude of biological properties, including suppression of tumor cell growth, reduction of malaria para-locus replication, alleviation of inflammation, and resistance to viral invasion (Feng et al., 2010; Wang et al., 2011). BA exhibits anti-pancreatic cancer effects by suppressing pancreatic cancer growth and inducing apoptosis through the stress-activated protein kinase/mitogen-activated protein kinase/nuclear factor-κB/signal transducer and activator of transcription 3/B cell lymphoma-2 (JNK/p38 MAPK/NF-κB/Stat3/Bcl-2) signaling pathway (Lu et al., 2021). However, few studies have been reported on BA in CC, moreover, the effects and its mechanism are still unclear.

With its innovative concept of multicomponent-multitarget-multipathway, network pharmacology integrates diverse databases and explores drug mechanisms by utilizing bioinformatics, which offers novel approaches to the study of complex Chinese medicine systems (Shao and Zhang, 2013; Zhou et al., 2020). Molecular docking is a pioneering technique based on computer simulation of structures to predict ligand-receptor interactions at the molecular level through docking means or to assess conformational relationships to identify compounds of therapeutic significance (Pinzi and Rastelli, 2019; Jakhar et al., 2020).

In this study, we intend to investigate the potential targets and related molecular mechanisms of BA for the treatment of colon cancer by means of network pharmacology and molecular docking. *In vitro* pharmacology experiments were conducted to confirm the reliability and accuracy of the network pharmacology investigation results, with a view to providing scientific evidence for the in-depth study and reasonable clinical application of BA.

## Materials and methods

### Materials

Bruceine A (BA) was supplied by Macklin, and its structure and purity (>98%) were characterized by nuclear magnetic resonance (NMR), high resolution mass spectrometry (HRMS) and high-performance liquid chromatography (HPLC). LY294002 and Acetylcysteine (NAC) were purchased from Chengdu Pukang Biotechnology Co., Ltd. and used without further purification. DMEM and 1,640 medium were purchased from Corning. 3-(4,5-dimethylthiazol-2-yl)-2,5-diphenyltetrazoliumbromide methyl thiazolyl tetrazolium (MTT), sodium dodecyl sulfate (SDS), dimethyl sulfoxide (DMSO), glycine, tris, and trypsin were obtained from Biofroxx. Fetal bovine serum (FBS) was supplied by Excell. The Annexin V-FITC/PI apoptosis detection kit, cell cycle detection kit, BCA kit, beyoclink™ Edu cell proliferation kit, penicillin, PMSF, mitochondrial membrane potential assay with IC-1, blotting grade and streptomycin were purchased from Beyotime. RIPA buffer, sodium dodecyl sulfate polyacrylamide gel electrophoresis (SDS-PAGE) and dual-color prestained protein maker were supplied by Epizyme. PVDF membranes were offered by immobilon. Antibodies against Akt, p-Akt, PI3K, p-PI3K, caspase-3/8/9, Bax, Bcl-2, *β*-actin and *β*-tubulin were purchased from Cell Signaling Technology. Antibodies against Cyclin A, Cyclin B, Cyclin D, Cyclin E, CDK1, CDK2, CDK4, CDK6, PARP/Cleaved-PARP, and horseradish peroxidase-conjugated secondary antibodies were offered by Immunoway. Enhanced chemiluminescence (ECL) kit was purchased from the US.

### Screening of potential targets

The 3D structure of the BA ligand was downloaded from the PubChem database (http://pubchem.ncbi.nlm.nih.gov/). and its targets were obtained from the Swiss Target Prediction Database (http://www.swisstargetprediction.ch/), the Pharmmapper Database (http://www.lilab-ecust.cn/pharmmapper/), and the SuperPred Database (http://prediction.charite.de/). These protein targets were uniformly normalized in UniProt (http://www.uni-prot.org/) after deduplication. The CC-related targets were acquired from GeneCards (http://www.GeneCards.org/) with a correlation score ≥10 and OMIM (https://OMIM.org/) with a disease score ≥0.8. The common targets between BA and CC were identified by jveen (http://jvenn.toulouse.inra.fr/).

### Protein-protein interaction (PPI) network construction

In order to analyze the common protein targets between BA and CC, they were input into the String database (https://string-db.org/) to generate a protein-protein interaction (PPI) network. Only PPI interaction for *homo sapiens* was considered and a confidence score >0.9 was requested. The TSV format of the PPI network was downloaded and imported into Cytoscape 3.9.1 software to construct the PPI network and obtain hub genes.

### GO and KEGG pathway enrichment analysis

Gene Ontology (GO) and Kyoto Encyclopedia of Genes and Genomes (KEGG) pathway analyses were performed to analyze the common protein targets between BA and CC by using the David database (https://david.ncifcrf.gov/). All results were screened at *p* < 0.05 and the top 20 genes were visualized as bar plots and bubble plots using the bioinformatics (http://www.bioinformatics.com.cn/).

### Component-Target-Pathway network construction

In order to analyze the complex associations among BA, CC, common targets, and pathways, a Component-Target-Pathway (C-T-P) network was constructed by Cytoscape 3.9.1 software.

### Molecular docking

The 3D structures of PI3K (1E8Q) and Akt (4gv1) were obtained from the PDB database (https://www.rcsb.org/), and the proteins were dehydrated and dephosphorylated using PyMoL software. The AutoDockTools-1.5.6 software was then used to conduct hydrogenation, calculate the total charge, conduct molecular docking and select the mode with the low binding free energy of the target proteins and bioactive components as the research object. Molecular binding energy calculation was used to determine the target protein and bioactive component binding affinity by AutoDock Vina (the results are considered credible when the docking binding free energy is lower than - 5 kJ/mol). Two- and three-dimensional images of the resulting structure were generated with LigPlot and PyMoL.

### Cell lines and cell culture

Human colon cancer HCT116 cells, mouse colon cancer CT26 cells and human normal colonic epithelial cells NCM460 were obtained from the Cell Bank of Type Culture Collection of Chinese Academy of Sciences. The cells were incubated in RPMI 1640/DMEM with 10% FBS, 1% penicillin and 1% streptomycin, and maintained in a 37°C incubator with 5% CO_2_.

### Cell viability assay

MTT assays were performed to assess the effect of BA on HCT116 and CT26 cells. In brief, cells were seeded in 96-well plates at appropriate cell density (2.5 × 10^3^ cells/well) and incubated for 24 h until they adhered. After treatment with BA in different concentrations and DMSO (0.1%) as a control group for 24 or 48 h, MTT solution (20 μL, 5 mg/mL) was added to each well, and cells were incubated in the dark for 2 h. Then the medium was removed, and crystals were dissolved in 150 μL DMSO. Absorbance at 490 nm was measured using a microplate reader. For additional inhibitor experiments, cells were treated with NAC (2 mM) and LY294002 (20 μM) in the absence and presence of BA (30 nM or 250 nM) for 48 h. The rest of steps were same as the MTT protocol described above.

### Cell proliferation assays

An EeyoClink™ Edu cell proliferation kit with alaxe fluor 555 was used to evaluate cell proliferation ability. HCT116 and CT26 cells were seeded in 24-well plates and incubated for 24 h until they adhered. After treatment with BA for 48 h, Edu, a nucleoside analog of thymidine, was incorporated into DNA during active DNA synthesis. Cells were then fixed and permeabilized, and Edu was detected through the click reaction. After labeling, the nuclei were stained with Hoechst 33,258. The experimental results were visualized by a confocal laser scanning microscope.

### Cell-cycle analysis

HCT116 and CT26 cells were separately inoculated into 6-well plates (4 × 10^4^ cells/well), and treated with different concentrations of BA for 48 h after cell adherence. Then cells were collected with trypsin, washed with PBS, and fixed in 70% ethanol overnight at 4°C. The fixed cells were centrifuged, washed, and resuspended in 500 μL buffer containing 10 μg/mL RNase and 25 μg/mL PI for 30 min in the dark. The final results were detected by flow cytometry.

### Apoptosis assays

HCT116 and CT26 cells were individually seeded into 6-well plates (4 × 10^4^ cells/well) and treated with different concentrations of BA for 48 h after cell adherence. Then cells were collected with trypsin, washed with PBS, and stained with 195 μL binding buffer which added 5 μL Annexin V-FITC solution and 10 μL PI solution. After staining, the samples were analyzed by flow cytometry.

### Detection of reactive oxygen species (ROS)

HCT116 and CT26 cells were treated with different concentrations of BA for 48 h after cell adherence. The treated cells were stained with DCFH-DA for 30 min. After labeled, cells were washed twice with ice-cold PBS, trypsinized, and harvested in cold PBS. The final results were detected by flow cytometry.

### Mitochondrial membrane potential (MMP) (ΔΨm) detection

HCT116 and CT26 cells were treated with different concentrations of BA for 48 h after cell adherence. Then cells were washed twice with PBS, trypsinized, suspended in JC-1, and incubated in the dark for 20 min at 37°C, followed by washing twice by JC-1 working solution and suspending in a basic medium. The final results were detected by flow cytometry.

### Cell migration assays

HCT116 or CT26 cells (4 × 10^4^ cells) in 400 μL serum-free medium containing different concentrations of BA were seeded into the upper chamber of a transwell, and 600 μL medium with 10% FBS was placed in the lower chamber. After incubating at 37°C for 24 h, cells on the upper membrane surface were scraped off. The invasive cells were fixed with 4% paraformaldehyde and stained with 0.1% crystal violet. The results were observed under an inverted microscope.

### Wound-healing migration assay

HCT116 or CT26 cells were seeded into 6-well plates and cultured until approximately 80% confluence in monolayer cultures. Artificial scratches were created by the end of a 200 μL pipette tip and the cell debris was washed with PBS. After incubating in serum-free DMEM/1,640 with the indicated concentration of BA for 24 h, cell migration was observed under a phase-contrast microscope at ×40 magnification field at 0 and 24 h post-induction of injury. The inhibition of migration caused by BA was measured and quantified with the computer-assisted microscope.

### Western blot analysis

Cells treated with different concentrations of BA, LY294002 (20 μM) and NAC (2 mM) alone or in combination for 24 h were washed with cold PBS and collected by RIPA-containing cocktail and PMSF on ice. The lysate was centrifuged by high-speed and low-temperature centrifugation. The BCA method was used to detect the protein concentration. Protein samples were respectively separated by 12.5% SDS-PAGE or 10% SDS-PAGE and transferred to PVDF membranes. The membranes were blocked with 5% nonfat dry milk for 2 h at room temperature before being incubated with the corresponding primary antibodies (1:1,000) in TBS-T overnight at 4°C. Then, the membranes were washed three times with TBS-T buffer and incubated with appropriate horseradish peroxidase-conjugated secondary antibodies at room temperature for 2 h. Finally, the protein bands were detected with an ECL kit, and the results of each band were quantified by ImageJ software.

### CETSA assay

After treatment of cells with the indicated concentrations of BA for 3 h, cells were collected with PBS containing protease inhibitors and transferred to 200 μL EP tubes. Cells were heat shocked at 45–61°C for 3 min, then cyclically lysed in liquid nitrogen to 25°C for 3 times, followed by centrifugation at 4°C, 12,000 rpm for 20 min, and finally loading buffer was added for standard Western blot operation.

### Statistical analysis

Data were analyzed by Prism 9.0 software (GraphPad, La Jolla, CA, United States) and expressed as mean ± standard deviation (SD). The differences between groups were analyzed using an unpaired *t*-test when only two groups were compared and a one-way analysis of variance when more than two groups were compared. Values of *p* < 0.05 were considered to be statistically significant.

## Results

### Potential BA targets and CC targets

**FIGURE 1:**
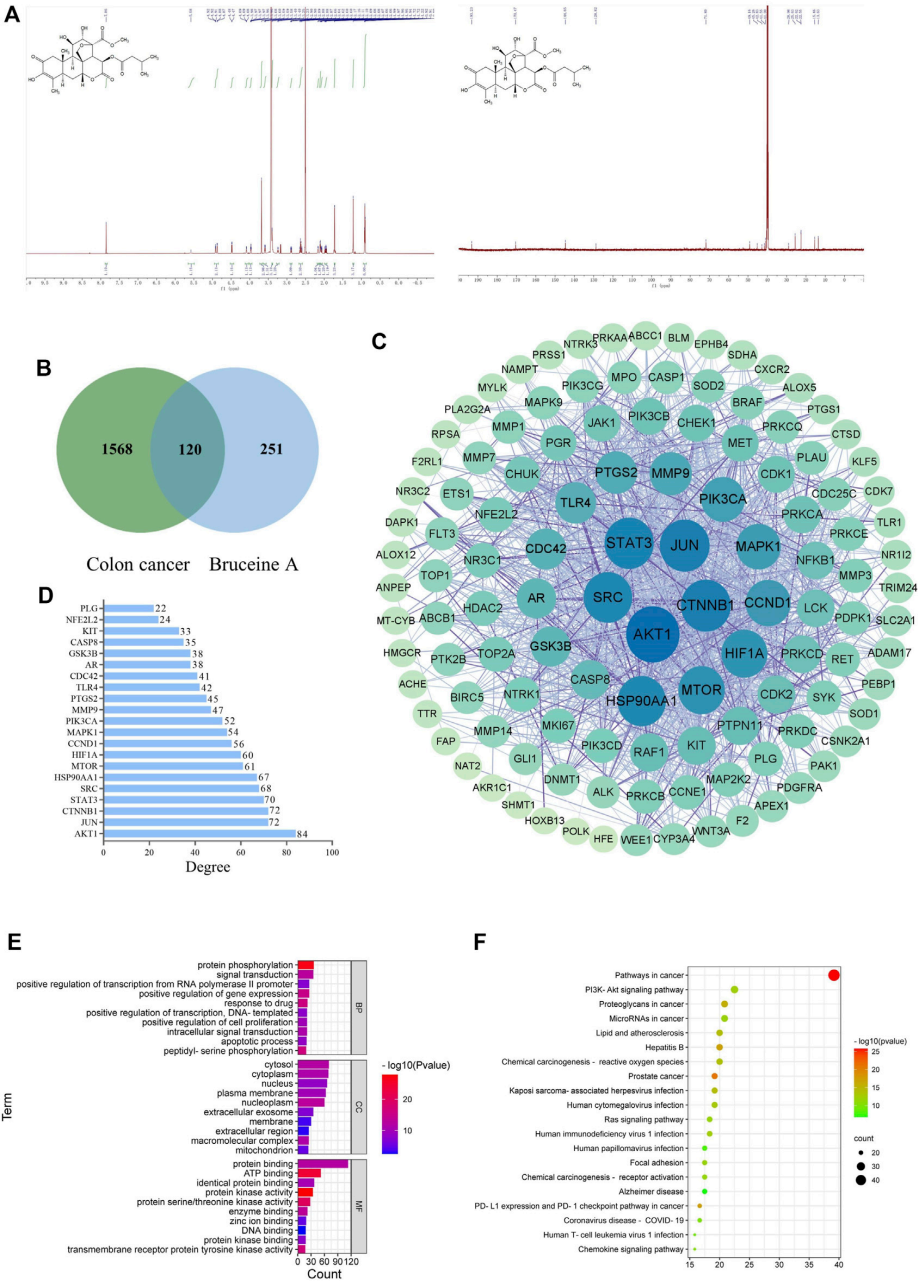
Network pharmacology analysis. **(A)** The chemical structure of Bruceine **(A,B)** Venn diagram of Bruceine A targets and colon cancer-related targets. **(C)** PPI network of common targets of BA and CC. The larger the node and the darker the color is, which means the higher the degree of correlation. **(D)** Bar plot showing the top 21 hub genes in the PPI network. **(E)** The top 20 enriched KEGG pathways of target genes. **(F)** GO analysis of target genes.

The chemical structure of BA was identified *via* ^1^H NMR and ^13^C NMR spectra (Figure 1A). After searching relevant targets in Swiss, Pharmmapper, and Superpred databases, a total of 371 potential targets of BA were collected by correction and elimination of duplicate items in UniProt database. Then 1,688 colon cancer-related targets were screened and collated from the GeneCards and OMIM databases. A grand total of 120 genes were identified by mutually matching the target genes of BA with those of colon cancer, implying that BA might perform the therapeutic effect of CC through these 120 target genes (Figure 1B).

### Establishment of PPI network for BA treatment of CC related targets

The 120 potential targets were submitted to the STRING database for analysis, and the target protein interaction data relationships were generated and visualized *via* Cytoscape 3. 8. 0 software to construct a PPI network for BC therapeutic CC, which consisted of five concentric circles with 119 nodes and 1,301 edges (Figure 1C). Further topological analysis revealed the presence of 21 key nodes with the degree centrality (DC), closeness centrality (CLCE) and betweenness centrality (BC) greater than the mean value (DC > 21.87, CLCE > 115.66, BC > 0.0044) (Figure 1D). Additionally, the top five moderate values were Akt1, JUN, CTNNB1, STAT3, and SRC, indicating that they performed a relatively prominent role in the overall network, and the mechanism of BA in CC treatment might be associated with the modulation of these genes.

### GO function and KEGG pathway enrichment of BA-CC common targets

The GO enrichment analysis of potential targets for BA treatment of CC was conducted through the DAVID database, resulting in a total of 498 notable items, which comprised 353 items relevant to biological processes (BPs), 58 items relevant to cellular components (CCs), and 87 items relevant to molecular functions (MFs). In Figure 1E, the top 10 items in terms of relevance were displayed, in which the potential therapeutic targets were primarily associated with three BPs: protein phosphorylation, signal transduction, and positive regulation of gene expression; three CCs: cytosol, cytoplasm, and nucleus; three MFs: protein binding, ATP binding, and identical protein binding. Moreover, a total of 55 remarkable pathways were identified from the KEGG enrichment results (*p* < 0.05), and the top 20 pathways were ranked as illustrated in Figure 1F, involving mainly PI3K/Akt, Ras, and PD-L1 expression and PD-1 checkpoint pathways associated with tumors. Combined with the results of PPI network analysis, Akt1 is speculated to be one of the critical targets while PI3K/Akt signaling pathway might be the key pathway in BA treatment of CC.

### Target-pathway network analysis

**FIGURE 2:**
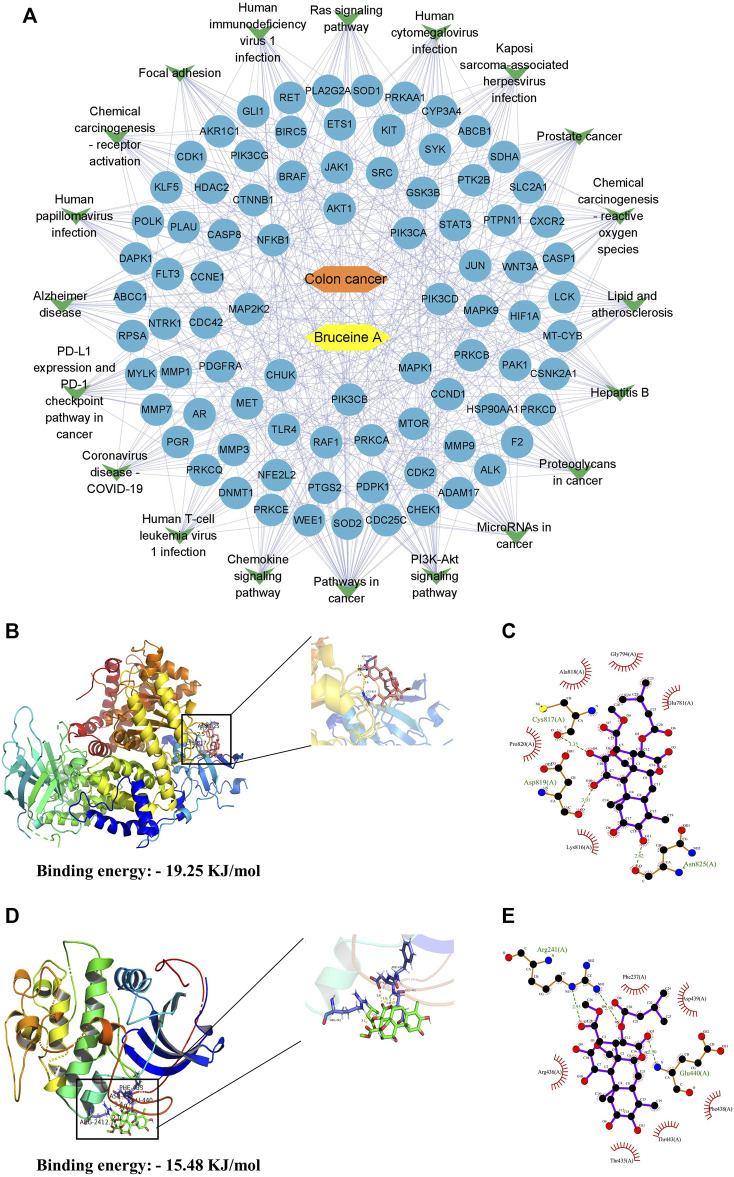
Results of molecular docking of targets with BA. **(A)** The compound-target-pathways network, yellow nodes indicate BA, orange nodes indicate colon cancer, blue oval nodes indicate the target nodes, and green triangle nodes indicate the corresponding pathways. **(B,C)** The molecular docking between BA and PI3K. 3D-diagram **(B)** and 2D-diagram **(C)** interaction analysis between BA and PI3K. **(D,E)** The molecular docking between BA and Akt. 3D-diagram **(D)** and 2D-diagram **(E)** interaction analysis between BA and Akt. BA molecule was displayed using a ball and stick model and the PI3K/Akt protein was displayed using a cartoon structural model.

The top 20 pathways corresponding to the targets were summarized and topologically analyzed by Cytoscape 3.9.1 software to build a “target-pathway” network (Figure 2A). The circle represents the target site and the V-shape represents the signaling pathway in the network. The network consisted of 107 nodes, of which the top 6 were PIK3CA, PIK3CB, PIK3CD, Akt1, MAP2K2, and MTOR, regulating multiple signaling pathways respectively, and might be the key targets for BA treatment of CC.

### Molecular docking of BA with critical targets

According to the correlation analysis of the KEGG pathway and PPI network, PI3K/Akt pathway might be the crucial pathway for BA treatment of CC. Therefore, PI3K and Akt in the PI3K/Akt pathway were molecularly docked with BA, respectively, employing AutoDockTools-1.5.6 software to evaluate the binding ability of BA to the target at the molecular level. The molecular docking results revealed that BA could bind to both PI3K and Akt proteins, with a binding energy of—19.25 kJ/mol to PI3K, mainly through three hydrogen bonds to ASN-825, Asp817 and CYS-817 (Figures 2B, C), and - 15.48 kJ/mol to Akt, forming three hydrogen bonds to Arg241 and Glu440 (Figures 2D, E). Conclusively, BA exhibited favorable binding activity to the target site, confirming that the predictions in this design were more reliable.

### BA inhibited HCT116 and CT26 cells proliferation

**FIGURE 3:**
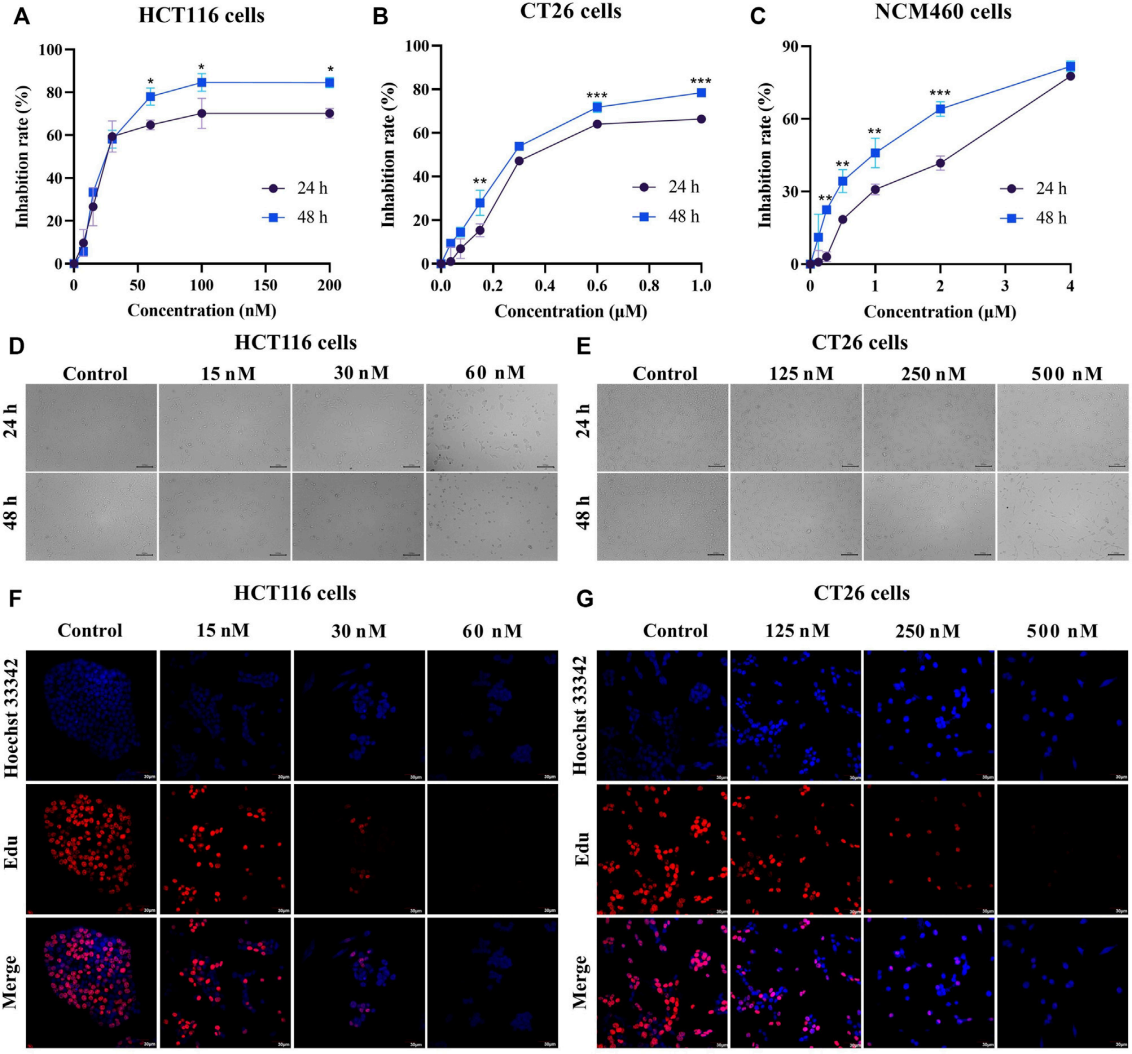
BA inhibited cell proliferation in HCT116 and CT26 cells. **(A–C)** The cell inhibition rate of HCT116 **(A)**, CT26 **(B)** and NCM460 **(C)** cells were treated with different concentrations of BA for 24 h or 48 h **(D,E)** The morphological changes of HCT116 **(D)** and CT26 **(E)** cells treated with BA for 24 h or 48 h **(F,G)** Representative images of Edu-labeled HCT116 **(F)** and CT26 **(G)** cells after 48 h of BA treatment. Red: Edu, Blue: Hoechst 33,324. *p < 0.05, **p < 0.01, ***p < 0.001, compared to the 24 h group.

To investigate the cytotoxic effects of BA, CC cell lines (HCT116 and CT26) were subjected to different concentrations of BA for 24 h and 48 h, respectively. The MTT results indicated that the proliferation of HCT116 and CT26 cells was dramatically suppressed after 24 and 48 h with different concentrations of BA in comparison with the control group, leading to statistically significant differences. The half-maximal inhibitory concentrations of BA at 48 h for HCT116 (Figure 3A) and CT26 (Figure 3B) cells were 26.12 ± 2.83 nM and 229.26 ± 12 nM, respectively. However, the proliferation inhibitory ability of BA on NCM460 cells was statistically lower than that of CC cells under the same concentration conditions (Figure 3C). Additionally, with the increasing concentration and time of drug administration, the cells progressively suffered from different degrees of deformation and contraction, as well as a continuous diminution in the number of cells (Figures 3D,E). Moreover, after 24 h of treatment with BA, the incorporated Edu. a new thymidine analog, was obviously reduced in the HCT116 (Figure 3F) and CT26 (Figure 3G) cells compared with the control group, which tended to decline with the increase of drug concentration. The above outcomes demonstrated that BA significantly suppressed the growth of CC cells and exclusively restrained the proliferation of HCT116 and CT26 cells at 60 nM and 500 nM, respectively.

### BA induced cell cycle arrest in HCT116 and CT26 cells

**FIGURE 4:**
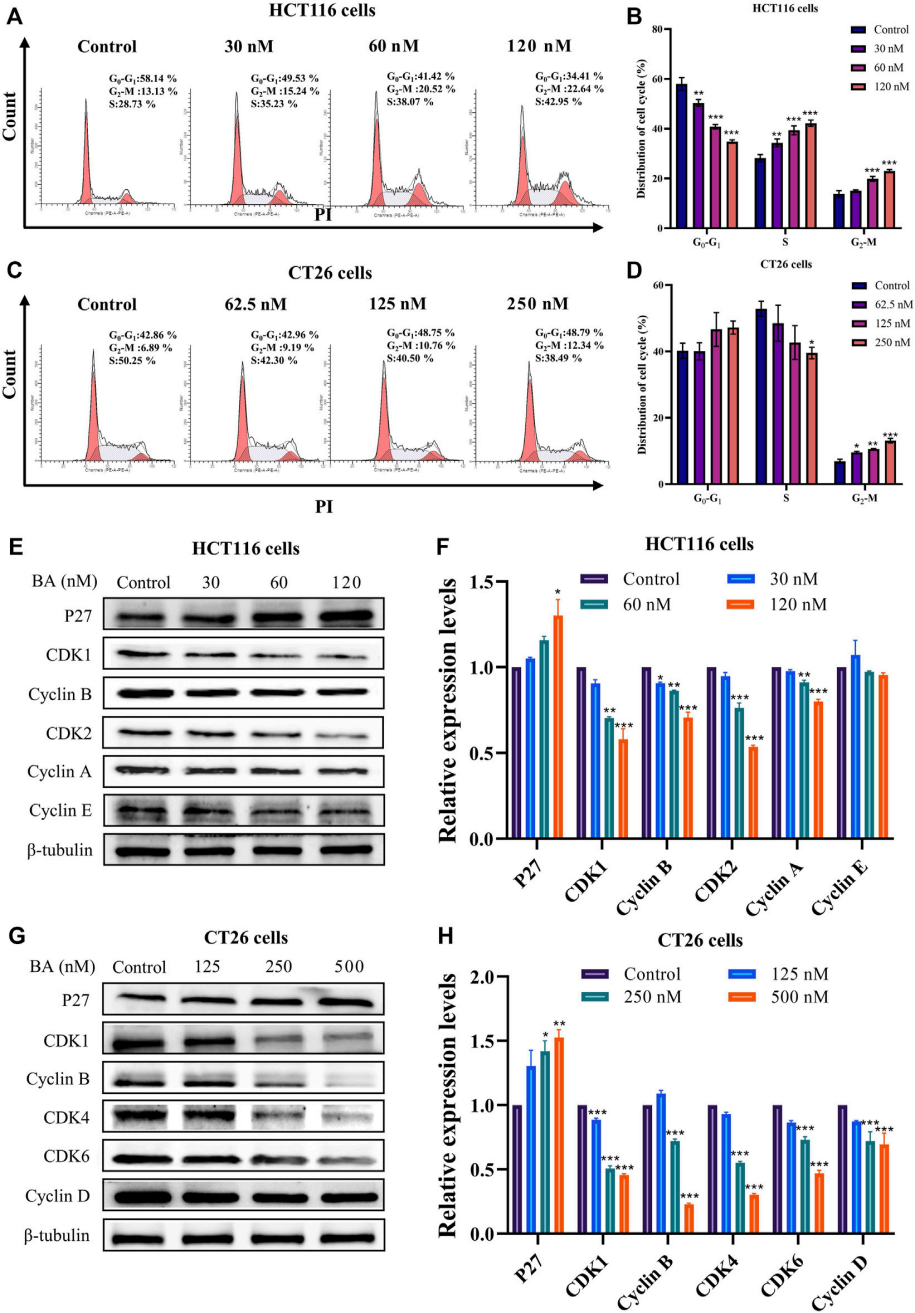
BA induced cycle arrest and modulated the expression of the cycle-related proteins in HCT116 and CT26 cells. **(A–D)** Cell cycle distribution of HCT116 **(A)** and CT26 **(C)** cells treated with BA. Statistical analysis of the percentage G0/G1, S and G2/M phases of HCT116 **(B)** and CT26 **(D)** cells treated with BA. **(E–H)** Western blot analysis for the expression of cycle related proteins in HCT116 **(E)** and CT26 **(G)** cells treated with BA. Statistical analysis of P27, CDK1, CDK2, Cyclin E, Cyclin B and Cyclin A levels in HCT116 cells **(F)**, and analysis of P27, CDK1, CDK4, CDK6, Cyclin B and Cyclin D levels in CT26 cells **(H)**. The protein levels were normalized to the control group. All data were representative of three independent experiments. *p < 0.05, **p < 0.01, ***p < 0.001, compared to the control group.

To validate whether the growth suppression effect of BA is attributed to disrupting the cell cycle, flow cytometry was employed to analyze the cell cycle distribution in HCT116 and CT26 cells. The results revealed that BA treatment induced a distinct arrest in S and G2/M phases on HCT116 cells (Figures 4A, B), and in G1 and G2/M phases on CT26 cells (Figures 4C, D), both in a concentration-dependent manner. In addition, to further explore the concrete mechanism of BA, Western blot assay was performed to determine the expression levels of cell cycle-related proteins. The levels of P27 were apparently escalated in BA-treated HCT116 and CT26 cells. The expression levels of CDK1, CDK2, Cyclin A, Cyclin B, and Cyclin E were significantly diminished in HCT116 cells (Figures 4E, F) after BA treatment for 24 h, and similarly, the expression levels of Cyclin D, Cyclin B, CDK2, CDK4, and CDK6 were dramatically decreased in CT26 cells (Figures 4G, H), both of which manifested concentration-dependent profiles. Taken together, the above results indicated that BA inhibited the proliferation of HCT116 and CT26 cells by activating the expression of P27 and thereby impacting the expression of downstream cell cycle-related proteins.

### BA engendered cell apoptosis in HCT116 and CT26 cells

**FIGURE 5:**
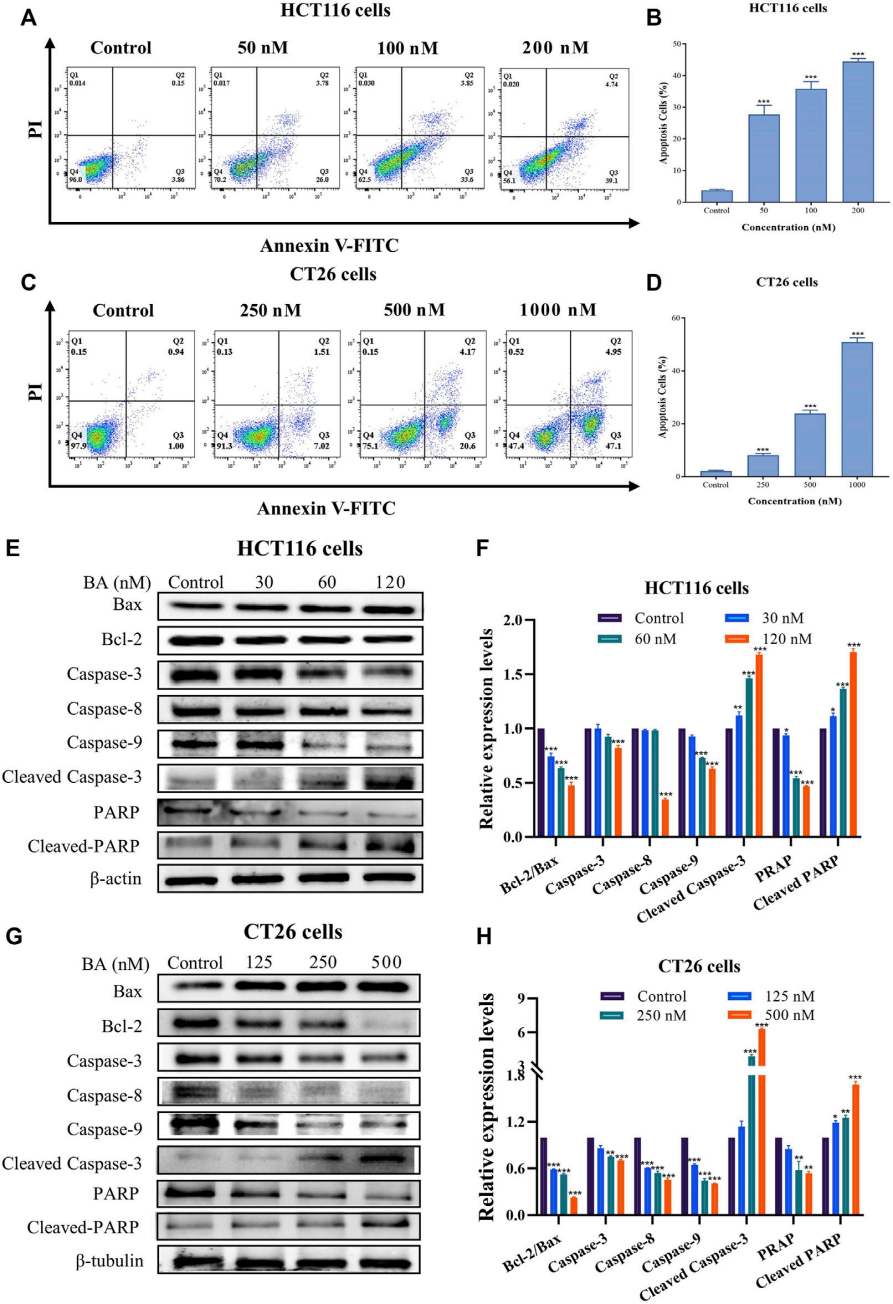
BA induced cell apoptosis in HCT116 and CT26 cells. **(A–D)** The level of apoptosis of HCT116 **(A)** and CT26 **(C)** cells treated with BA was assessed by Annexin V-FITC/PI double staining. Statistical analysis of the apoptosis rate of HCT116 **(B)** and CT26 **(D)** cells treated with BA. **(E–H)** The protein expression levels of Bax, Bcl-2, PARP, caspase-3/8/9, cleaved-RARP and cleaved-caspase-3 in BA-treated HCT116 **(E)** and CT26 **(G)** cells were measured by western blot, and *β*-actin or *β*-tubulin was served as a loading control. The relative protein levels of these proteins of HCT116 **(F)** and CT26 **(H)** cells were statistically analyzed, and the protein levels were normalized to the control group. **p < 0.01, ***p < 0.001, compared to the control group.

To thoroughly investigate the effect of BA on the physiological function of HCT116 and CT26 cells, the apoptotic activity of HCT116 and CT26 cells after BA treatment was determined by Annexin V/PI double staining method. In comparison with the control group, BA prominently enhanced the early apoptosis rate in both HCT116 and CT26 cells. The apoptosis rate increased from 4.01% to 29.78% at the BA administration concentration of 50 nM in HCT116 cells (Figures 5A, B) and raised from 1.94% to 24.77% at the BA administration concentration of 0.5 μM in CT26 cells (Figures 5C, D). To explore the mechanism of BA-induced apoptosis in HCT116 and CT26 cells, we further investigated whether the mitochondrial apoptotic pathway was involved in the regulation of BA-induced apoptosis. Western blotting revealed that BA treatment significantly declined the expression levels of Bcl-2, PARP and caspase 3/8/9, and augmented the expression levels of Bax, cleaved-PARP and cleaved-caspase 3 in a dose-dependent manner in HCT116 (Figures 5E, F) and CT26 cells (Figures 5G, H). The above results indicated that the apoptosis of CC cells induced by BA might be through the mitochondrial apoptosis pathway.

### BA inhibited migration of HCT116 and CT26 cells

**FIGURE 6:**
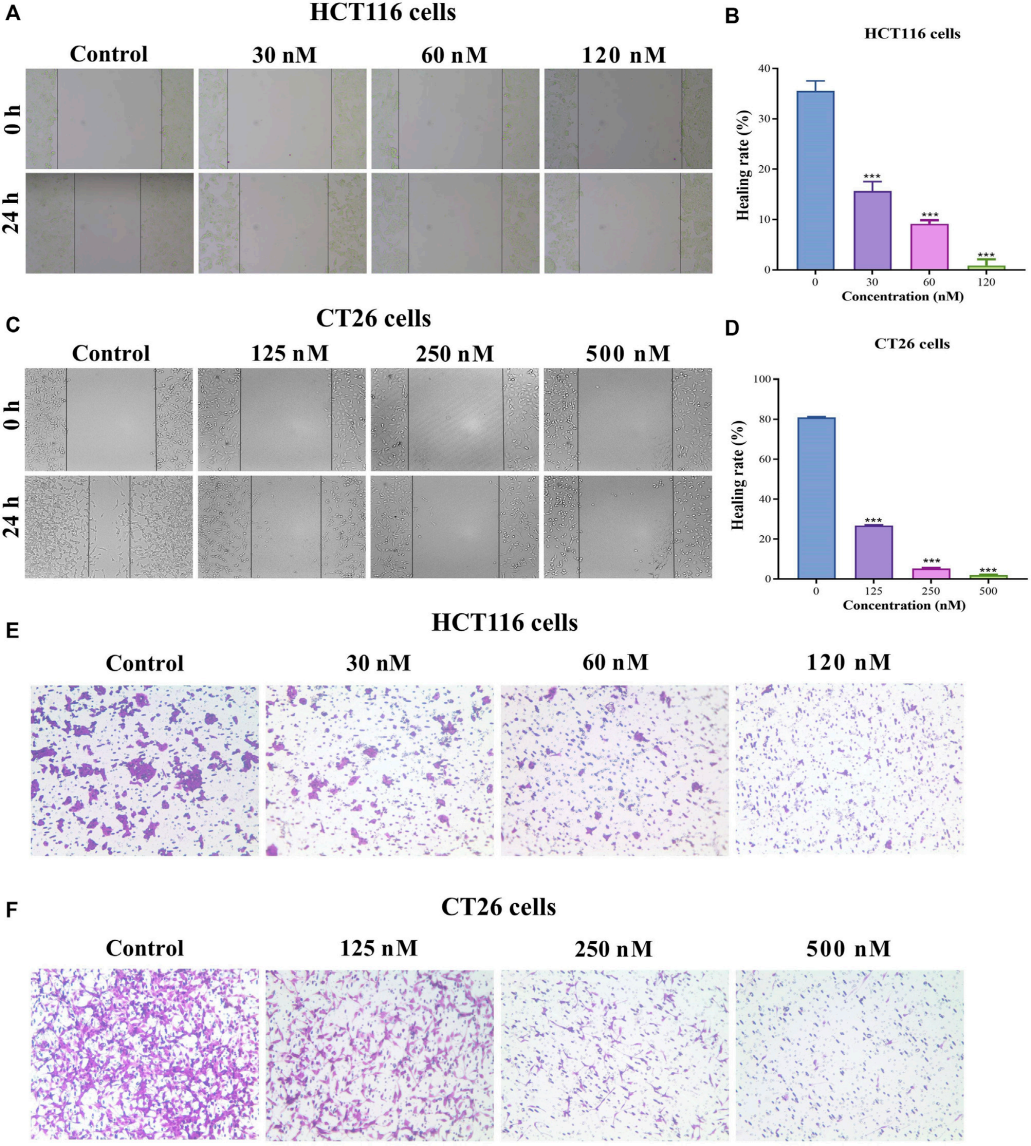
BA inhibited the migration of HCT116 and CT26 cells. **(A–D)** Cell migration in HCT116 **(A)** and CT26 **(C)** cells was determined by wound-healing assay. The photos represent cell migration under a microscope at ×100 magnification. The wound healing rate of HCT116 **(B)** and CT26 **(D)** was analyzed by ImageJ. **(E,F)** Cell migration in HCT116 **(E)** and CT26 **(F)** cells was determined by transwell migration assay. ***p < 0.001, compared to the control group.

To comprehensively explore whether BA exerts an inhibitory effect in CC progression, both wound-healing migration and Transwell migration assays were employed. The results indicated that BA treatment effectively suppressed wound closure in a dose-dependent manner. Moreover, the majority of cells in the vehicle group migrated to the scraped line, whereas the number of migrated cells was obviously diminished in the BA-treated group, and 120 nM and 0.5 μM of BA could inhibit the migration of about 99% HCT116 (Figures 6A, B) and CT26 (Figures 6C, D) cells, respectively. In the Transwell migration assay, the horizontal migration capability of HCT116 (Figure 6E) and CT26 (Figure 6F) cells was notably restrained by BA, and the number of migrating cells in the BA-treated group was clearly lower than that in the control group. The above results illustrated that BA possessed the potential to suppress the migration of CC cells.

### BA modulated the ROS levels in HCT116 and CT26 cells

**FIGURE 7:**
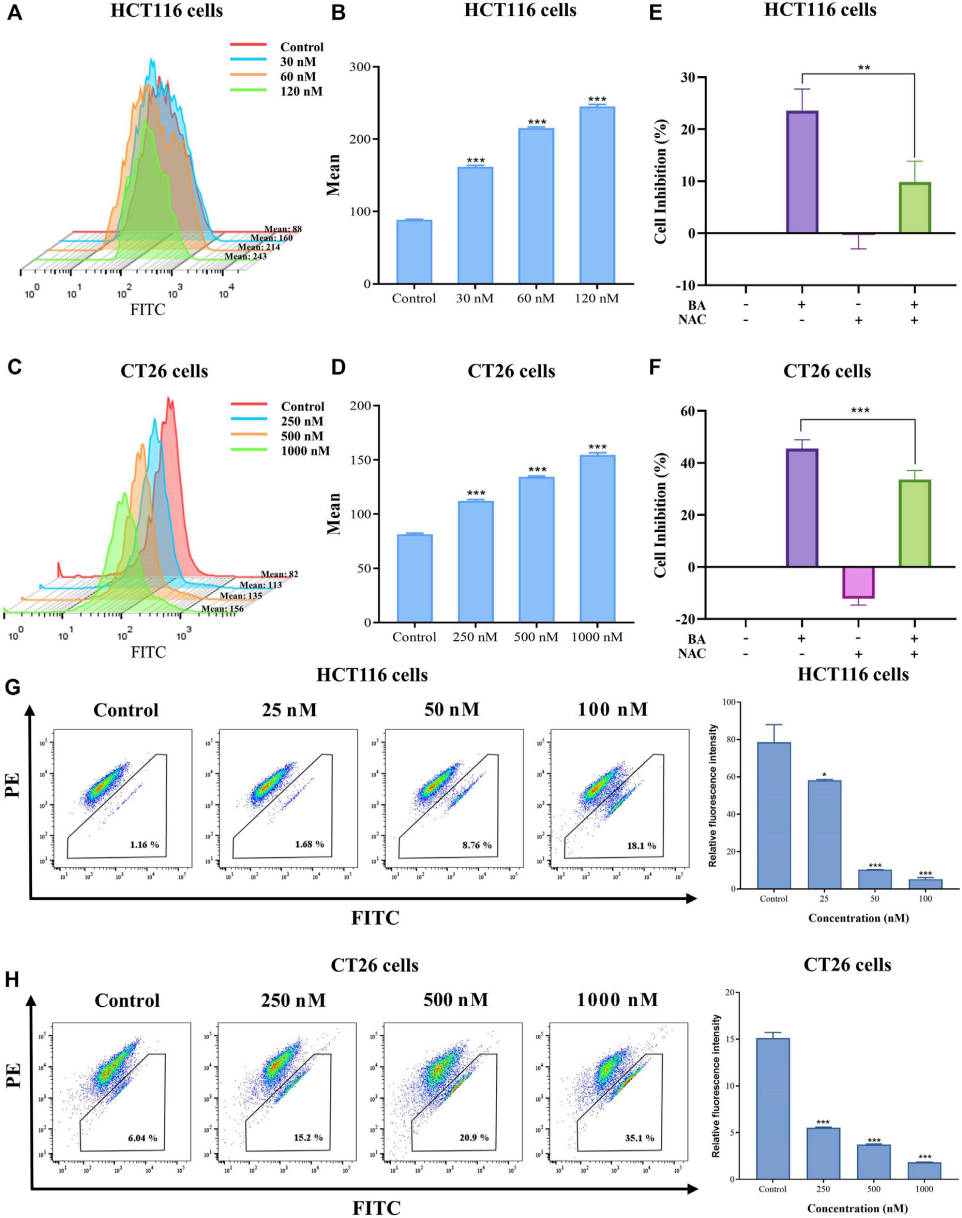
BA induced ROS production and collapsed MMP. **(A–D)** BA induced ROS production in HCT116 **(A)** and CT26 **(C)** cells Statistical analysis of the median fluorescence intensity of ROS in HCT116 **(B)** and CT26 **(D)** cells treated with BA. **(E,F)** The cell inhibition rate of HCT116 **(E)** and CT26 **(F)** cells were treated with BA and/or NAC (2 mM) for 48 h **(G,H)** The changes in MMP in BA-treated HCT116 **(G)** and CT26 **(H)** cells. **p < 0.01, ***p < 0.001, compared to the control group.

ROS performs an essential function in the induction of apoptosis in both physiological and pathological situations. Previous reports showed that the derivative of BA evoked ROS production and initiated the DNA damage response, leading to lung cancer cell apoptosis (Fan et al., 2020a). Therefore, we inspected whether BA provokes ROS accumulation utilizing cell-permeable dyes in colon cancer cells. As illustrated in Figures 7A, B, the median peak profiles were shifted to the right in BA-treated cells, implying that BA administration induced an elevated level of ROS in HCT116 and CT26 cells as compared to untreated cells. Moreover, the intracellular ROS levels were remarkably increased in BA-treated HCT116 and CT26 cells in an administration concentration-dependent manner, implicating that ROS production might be involved in BA-induced apoptosis in CC cells. To investigate whether the enhancement of ROS is involved in BA-induced apoptosis in CC cells, NAC, an inhibitor of ROS, was employed to pretreat CC cells. According to the results, the proliferation viability of HCT116 and CT26 cells was augmented by NAC, and the inhibitory effect of BA on the proliferation of HCT116 and CT26 cells was obviously diminished when 2 mM of NAC was treated with CC cells (Figures 7E, F), which indicated that ROS generation might be associated with BA-induced apoptosis in CC cells.

### BA collapsed MMP (∆Ψm) in HCT116 and CT26 cells

As mitochondria perform a crucial function in apoptosis, cascade apoptosis mediated by mitochondria is generally associated with the collapse of MMP due to leakage of pro-apoptotic factors. JC-1 staining, typically employed to measure changes in MMP, manifests a red fluorescence responding to high MMP in a blank control. Green fluorescence was dramatically visible in HCT116 and CT26 cells after being exposed to various concentrations of BA for 48 h. The variation from red fluorescence to green fluorescence indicated that BA induced a decrease in MMP in a dose dependent manner, implying that the diminution in mitochondrial membrane potential might be involved in BA-induced apoptosis of CC cells.

### BA exerted anticancer effects *via* regulating PI3K/Akt pathway in HCT116 and CT26 cells

**FIGURE 8:**
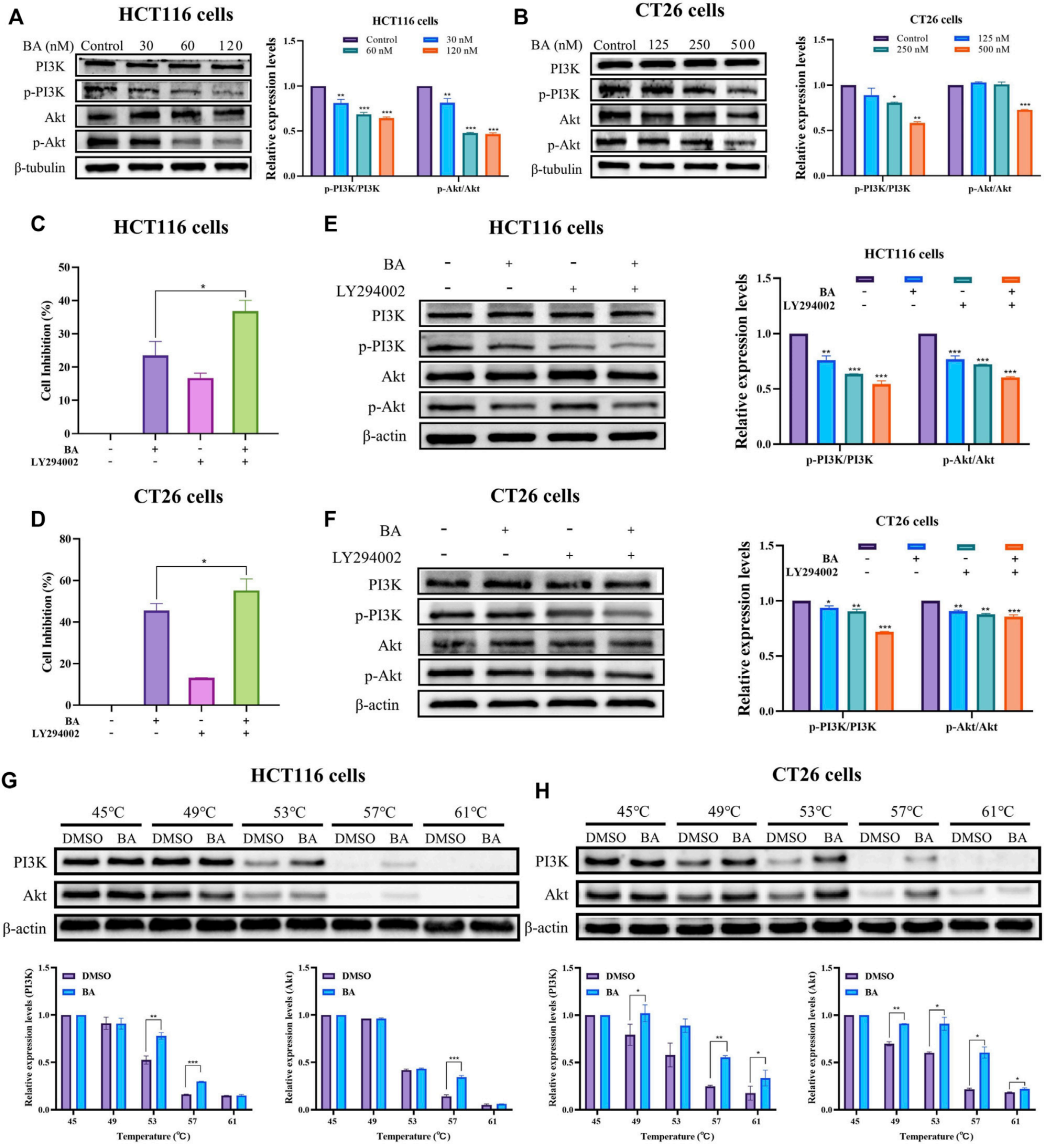
BA inhibited the PI3K/Akt signaling pathway. **(A,B)** The PI3K/Akt pathway related protein expression levels of HCT116 **(A)** and CT26 **(B)** cells treated with BA were measured by western blot, and *β*-tubulin served as a loading control. **(C,D)** The cell inhibition rate of HCT116 **(C)** and CT26 **(D)** cells were treated with BA and/or LY294002 for 48 h **(E,F)** The PI3K/Akt pathway related protein expression levels of HCT116 **(E)** and CT26 **(F)** cells treated with BA and/or 2 LY294002 (20 μM) were measured by western blot, and *β*-tubulin or *β*-actin served as a loading control. **(G,H)** Western blotting intensity and standardized statistics of PI3K and Akt in HCT116 **(G)** and CT26 **(H)** cells with or without BA in CETSA test. *p < 0.05, **p < 0.01, ***p < 0.001, compared to the control group.

The P13K/Akt pathway is extensively present in a variety of cancer cells and performs an imperative regulatory role in cell growth, proliferation, and angiogenesis. The previous network pharmacology results have predicted that the PI3K/Akt signaling pathway was the supreme target enrollment, implying that it might be a critical pathway for BA treatment of CC. Western blot results revealed that BA attenuated the phosphorylation of PI3K and inhibited Akt phosphorylation in a dose-dependent manner in both cell lines. Further data analysis showed that the BA treatment group dramatically enhanced the protein expression ratio of PI3K/p-PI3K and Akt/p-Akt as compared to the control groups (Figures 8A, B). To further validate the involvement of PI3K/Akt pathway in the anti-CC effect of BA, a control treatment was performed with 20 μM of LY294002, a PI3K inhibitor suppressing PI3K activation. The MTT results revealed that treatment with 20 μM LY294002 alone could suppress the proliferation viability of HCT116 and CT26 cells, whereas the proliferation inhibitory activity was further potentiated when co-administered with 30 nM or 250 nM BA to HCT116 or CT26 cells (Figures 8C, D), which confirmed the proliferation inhibitory effect of BA in accordance with LY294002. Meanwhile, Western blot further examined the effects of LY294002 and BA on PI3K protein expression, demonstrating that BA exhibited a PI3K inhibitory effect consistent with LY294002, and PI3K expression was further dramatically decreased by combined treatment with LY294002 and BA (Figures 8E, F). To further determine whether BA interacts directly with PI3K/Akt, the CETSA assay was introduced for the validation of label-free targets. The thermal stability of PI3K and Akt after BA treatment was enhanced compared with the control (DMSO), and the results indicated that BA could existence a positive interaction with PI3K and Akt, further supporting the existence of binding energy between the computer simulated BA and the target proteins (Figures 8G, H).

Taken together, the outcomes demonstrated that the suppression of cell proliferation and apoptosis by BA might be correlated with the downregulation of PI3K/Akt pathway.

## Discussion

CC is the most common malignant tumor worldwide, with the incidence and death rate increasing annually, which seriously endangers the patients’ healthcare and life quality. Although chemotherapy and drug combination therapy are generally adopted in the clinical treatment of advanced CC, toxin accumulation and drug resistance are the primary demerits in these treatments (Li et al., 2022a). Therefore, it is urgent to seek new anti-tumor drugs with high efficiency and low toxicity.

Chinese herbs and their extracts have demonstrated great potential in the treatment of various diseases, especially neoplasms (Yang and Wu, 2015; Fan et al., 2020b; Li et al., 2022b; Fu et al., 2022; Hamza et al., 2023). BA is a species of flavonoids extracted from *C. ophiopogonis* seeds, and most recent studies have confirmed that BA could inhibit the proliferation of human NSCLC H460 cells and notably induce their apoptosis (Tan et al., 2019). However, there are only a few reports on its pharmacological properties, in particular, its anticancer effects and mechanism. Therefore, the mechanism of BA against CC was investigated through systematic network pharmacology and molecular docking in this study, and validated by relevant *in vitro* assays.

From Swiss, Pharmmapper, and Superpred databases, a sum of 371 therapeutic targets of BA were obtained, and a cumulative number of 1688 CC targets were identified through GeneCards and OMIM databases, resulting in 120 potential targets of BA for the treatment of CC. Topological analysis was performed by an established PPI network, and 21 key nodes were acquired, among which the core targets were Akt1, JUN, CTNNB1, STAT3 and SRC. The three enriched entries of common gene targets by GO enrichment analysis in terms of biological processes, cellular components, and molecular functions were 353, 58 and 87, respectively, which might be involved in immune response, apoptotic processes, protein binding, transcription factor binding, etc. The enrichment analysis of the KEGG pathway revealed that the common targets were enrolled in three major tumor-related signaling pathways, involving mainly PI3K/Akt, Ras, and PD-L1 expression and PD-1 checkpoint pathways, among which PI3K⁃Akt pathway exhibited a high level of enrichment and might be crucial for BA in treating colon cancer.

The PI3K/Akt pathway, a pivotal signaling pathway, performs an essential role in the regulation of cell growth, proliferation and apoptosis. PI3K activation allows stimulation of Akt through phosphorylation of the thr308 site of Akt, which regulates the Bcl-2 protein family to control apoptosis (Vier et al., 2016). Then the activated Akt phosphorylates the ser136 site of the Bad, leading to the release of Bcl-2, which binds to Bax to form a dimer, or phosphorylates the ser184 site of the Bax, binding to Bcl-2 to form a dimer (Blume-Jensen et al., 1998). Additionally, the Bcl-2 protein family is classified into a subfamily of Bax proteins promoting apoptosis and a subfamily of Bcl-2 proteins suppressing apoptosis. By regulating the ratio of pro-apoptotic proteins to anti-apoptotic proteins, especially the ratio of Bax/Bcl-2, apoptosis could be promoted or suppressed. In our study, BA promotes apoptosis in CC cells by suppressing the expression of the anti-apoptotic protein Bcl-2 and facilitating the apoptotic protein Bax, one of the downstream regulatory proteins of Akt. Caspases as a family of proteases serve an important function in the process of apoptosis. Activated caspase-9 (cleaved caspase-9) activates the key enzyme of apoptosis, caspase-3, which further enables subsequent apoptotic signals. Caspase-3 activation requires the production of active cleaved caspase-3 from inactive full-length caspase-3, thus caspase-3 activation is often considered as an essential indicator of apoptosis (Liang et al., 2002; Fan et al., 2005). BA suppressed the expression of caspase-3/8/9 and PARP, and promoted the cleaved caspase-3 and cleaved PARP expression, implicating the involvement of the mitochondrial apoptotic pathway in BA treatment of CC. Apoptosis in the mitochondrial pathway is regulated by the Bcl-2 family, which triggers alterations in MMP by escalating the permeability of the mitochondrial membrane, thereby regulating the amount of mitochondrial apoptotic proteins released. Studies in HCT116 and CT26 cells confirmed that BA promoted the decrease of mitochondrial membrane potential and accelerated apoptosis through the mitochondrial pathway. The activated Akt promotes the phosphorylation of cyclin-inhibitory proteins P21 and P27, rendering them incapable of binding and inhibiting the action of the cyclin/CDK complex, with the promotion of tumor cell proliferation. Previous studies have demonstrated that inhibition of the PI3K/Akt pathway diminishes Cyclin B1 and CDK1 expression levels, thereby leading to the arrest of CC cells and bladder cancer cells in the G2/M phase (Wang et al., 2017; Bhosale et al., 2021). Our results showed that BA suppressed the expression of Cyclin B, Cyclin D, CDK4, CDK6, and CDK1, thereby inhibiting the proliferation of CT26 cells in G1 and G2 phases, and arrested the cell cycle of HCT116 cells in S and G2 phases by repressing the expression of Cyclin A, Cyclin D, Cyclin E, CDK2, and CDK1. The results from molecular docking verification between BA and the targets revealed that BA displayed superior affinity for the targets, and the binding sites all contained stable hydrogen bonds with stable conformation and strong binding capability, further validating the accuracy of the predictions of this study and the reliability of *in vitro* experiments. CETSA has recently emerged as a powerful tool for elucidating the binding affinity of drugs to their target proteins (Jensen et al., 2015). The thermal stability of PI3K/Akt was detected to be dramatically augmented in BA-treated CC cells, thus providing an experimental argument that PI3K/Akt are the potential target proteins of BA.

ROS are in biological terms a natural by-product of normal oxygen metabolism with a vital role in cellular signal transduction and homeostasis (Sauer et al., 2001). Nevertheless, excessive accumulation of ROS could induce protein oxidation, lipid peroxidation, cellular DNA damage, and eventually cell death or apoptosis (Kumar et al., 2019). The present results demonstrated that BA dramatically elevated intracellular ROS levels in HCT116 and CT26 cells, induced caspase cascade activation, promoting apoptosis in CC cells.

Taken together, the above results demonstrated that BA suppressed colon cancer cell proliferation and promoted apoptosis by triggering a cascade reaction through accumulation of ROS and inhibition of PI3K/Akt pathway. Since the mechanism of BA against CC may not only affect the PI3K/Akt pathway, but also involve several other signaling pathways, related studies remain to be further explored.

## Conclusion

Based on network pharmacology, molecular docking technology, and *in vitro* relevant experiments, BA was found to induce apoptosis, inhibit proliferation and suppress migration of CC cells through suppression of PI3K/Akt pathway. These results suggest that BA is a potential drug candidate for the treatment of CC.

## Data Availability

The original contributions presented in the study are included in the article/supplementary material, further inquiries can be directed to the corresponding authors.
