# Supplementary material for: Bruceine a exerts antitumor effect against colon cancer by accumulating ROS and suppressing PI3K/Akt pathway
Source: Front Pharmacol. 2023 Mar 28;14:1149478. doi: 10.3389/fphar.2023.1149478 (PMC10086190; doi:10.3389/fphar.2023.1149478)
Supplement: Supplementary file 1 [file DataSheet1.pdf]

Bruceine A exerts antitumor effect against colon cancer by accumulating ROS and suppressing PI3K/Akt pathway

Chaozheng Zhang[1, (†)], Yuening Cao[1, (†)], Yi Zuo[1, (†)], Hongbin Cheng[1, 4], Changqun Liu, Bo Ren[1], Yun Deng[1], Maolin Wang[2, 3, *], Jun Lu[1, *]

1. State Key Laboratory of Southwestern Chinese Medicine Resources, School of Pharmacy, Chengdu University of Traditional Chinese Medicine, Chengdu, China;

2. Clinical Research Center, The First Affiliated Hospital of Shantou University Medical College, Shantou, Guangdong Province, China;

3. Department of Physiology, School of Basic Medical Sciences, Shenzhen University, China;

4. Hospital of Chengdu University of Traditional Chinese Medicine, Chengdu, China;

*** Correspondence:** Jun Lu, ljaaa111@163.com; Maolin Wang, wangml1240@163.com.

[(†)] These authors contributed equally to this work and share first authorship.

A

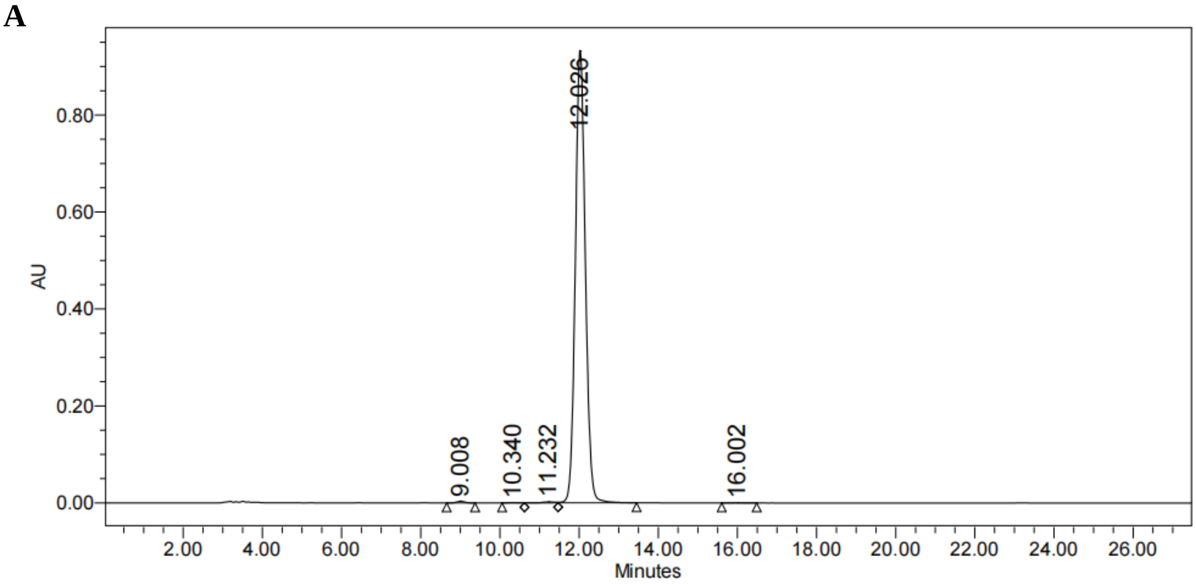

	RT	Area	% Area	Height
1	9.008	46813	0.28	3448
2	10.340	11434	0.07	698
3	11.232	44928	0.27	1918
4	12.026	16350449	99.32	934296
5	16.002	8704	0.05	393

B

Display Report

Analysis Info

Analysis Name	D:\Data\MCW-Data\HPLC-MS-Data\20230302\BA_21_1_6888.d	Acquisition Date	3/2/2023 10:05:54 AM
Method	HPLC - MS - positive.m	Operator	Demo User
Sample Name	BA	Instrument	timsTOF 1844426.00130
Comment			

Acquisition Parameter

Source Type	ESI	Ion Polarity	Positive	Set Nebulizer	2.0 Bar
Focus	Not active	Set Capillary	4500 V	Set Dry Heater	220 °C
Scan Begin	50 m/z	Set End Plate Offset	-500 V	Set Dry Gas	8.0 l/min
Scan End	1300 m/z	Set Collision Cell RF	400.0 Vpp	Set Divert Valve	Waste

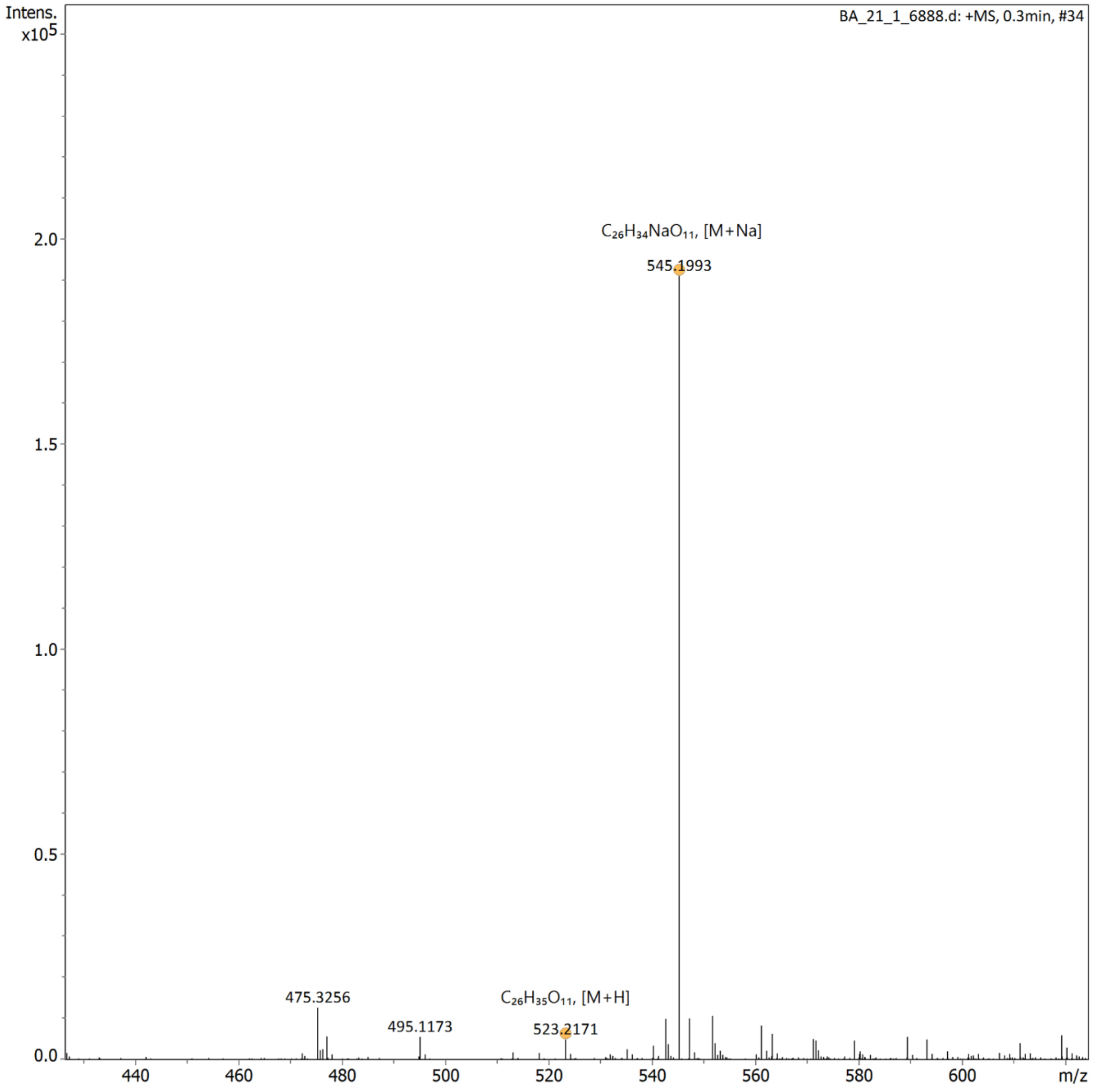

Bruker Compass DataAnalysis 5.3 printed: 3/2/2023 10:39:42 AM by: demo Page 1 of 1

SI Figure 1 HPLC（**A**） and HRMS（**B**） spectra of BA.

A

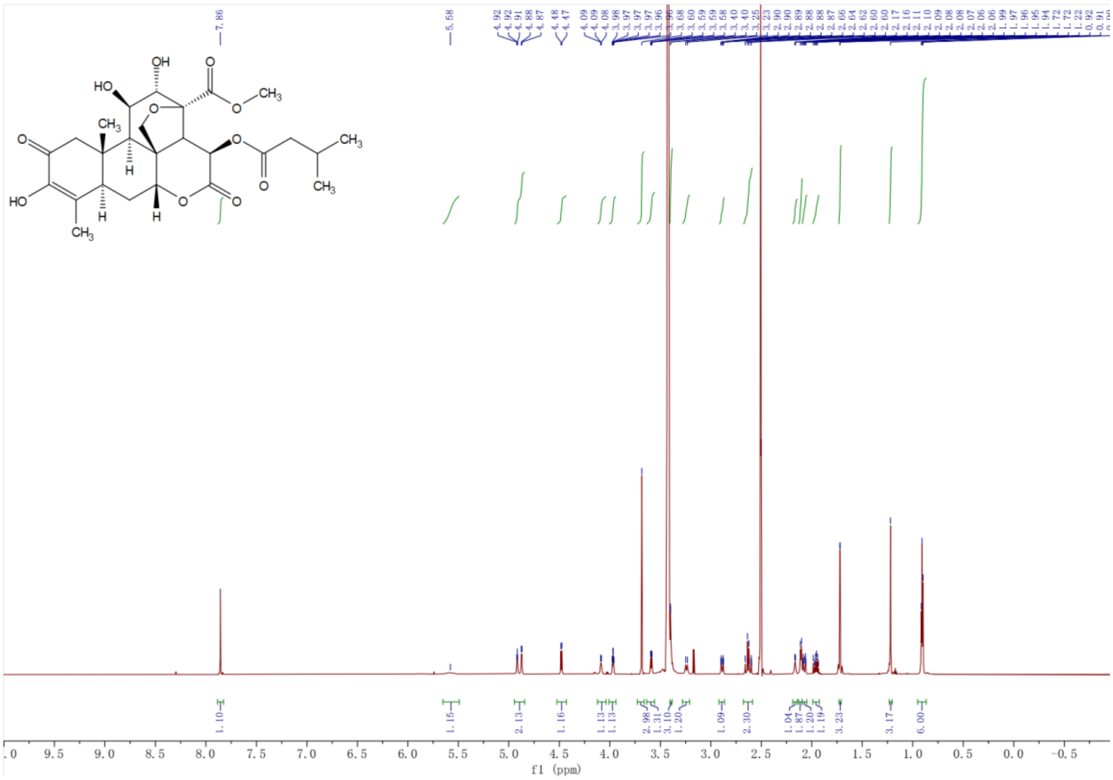

f1 (ppm)

B

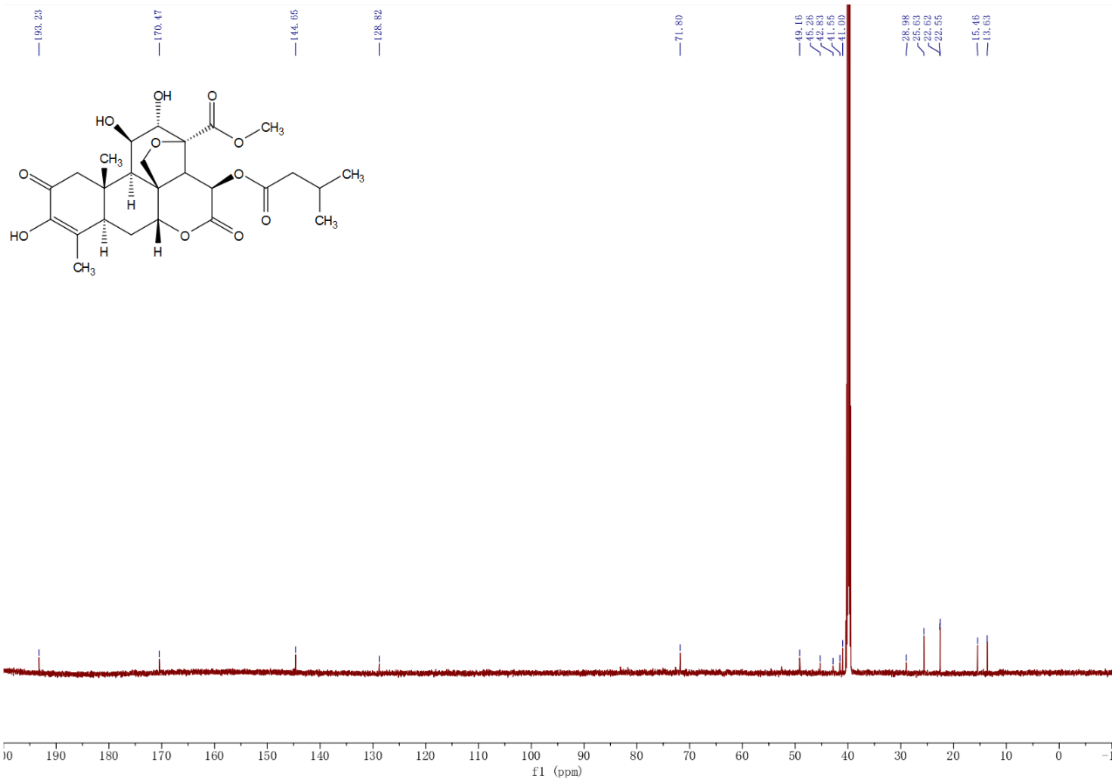

193.23
170.47
144.65
128.82
71.80
49.16
45.26
42.83
41.55
41.00
28.98
25.63
22.62
22.55
15.46
13.63
f1 (ppm)

SI Figure 2 ^{1}H-NMR and ^{13}C-NMR spectra of Bruceine A. （**A**） **^{1}H NMR (700 MHz, DMSO-*d_6*)** δ 7.86 (s, 1H, 3-OH), 5.58 (s, 1H, H-15), 4.95 – 4.84 (m, 2H, H-7, H-20a), 4.48 (d, *J* = 7.3 Hz, 1H, H-11), 4.12 – 4.04 (m, 1H, H-20b), 3.97 (td, *J* = 4.9, 1.6 Hz, 1H, H-12), 3.68 (s, 3H, 22-OMe), 3.59 (dd, *J* = 7.4, 1.7 Hz, 1H, 11-OH), 3.40 (m, 2H, H-9, H-14,), 3.24 (d, *J* = 13.0 Hz, 1H, H-1b), 2.89 (dt, *J* = 13.2, 2.3 Hz, 1H, H-1a), 2.68 – 2.59 (m, 2H, H-5, H-2'b), 2.16 (d, *J* = 4.9 Hz, 1H, 12-OH), 2.11 (d, *J* = 7.3 Hz, 2H, H-2'a, H-3'), 2.07 (dt, *J* = 14.8, 3.0 Hz, 1H, H-6b), 1.99 – 1.93 (m, 1H, H-6a), 1.72 (d, *J* = 1.9 Hz, 3H, 18-Me), 1.22 (s, 3H, 19-Me), 0.91 (t, *J* = 6.1 Hz, 6H, 4'/5'-Me). （**B**） **^{13}C NMR (176 MHz, DMSO-*d6*)** δ 193.23 (C-2), 170.47 (C-16, C-21, C-1'), 144.65 (C-3), 128.82(C-4), 82.90(C-7), 80.76(C-13), 71.8(C-11, C-20), 49.16(C-22, C-14), 45.26(C-9), 42.83(C-2'), 41.55(C-5), 41.00(C-10), 28.98(C-6), 25.63(C-3'), 22.62(C-5'), 22.55(C-4'), 15.46(C-19), 13.63(C-18). Based on the above data, The compound was identified as bruceine A, which was consistent with the reported litera ture values.
